# Effect of transport stress on apoptosis and autophagy in goat lung cells

**DOI:** 10.3389/fvets.2025.1585008

**Published:** 2025-07-11

**Authors:** Yu Zhuo, Yunhai Hu, Yangshan Jin, Tian Ye, Yanzhen Yang, Ben Liu, Wenya Zheng, Songlin Ding, Xue Yang, Lucheng Zheng, Wei Hu, Manxin Fang, Wanting Yi, Wenjing Xing

**Affiliations:** ^1^College of Life Science and Resources and Environment, Yichun University, Yichun, China; ^2^Yichun University Research Center for Traditional Chinese Veterinary Medicine and Animal Embryo Engineering Technology, Yichun, China; ^3^Engineering Technology Research Center of Jiangxi Universities and Colleges for Selenium Agriculture, Yichun, China

**Keywords:** transport stress, apoptosis, autophagy, Bcl-2/Bax, PINK1/Parkin

## Abstract

**Introduction:**

Road transportation exposes goats to thermal, mechanical, and microbial stressors that can compromise their welfare by triggering pulmonary apoptosis and autophagy processes associated with tissue damage and immunosuppression.

**Methods:**

To explore potential biomarkers for transport-related welfare assessment, this study analyzed lung tissues from nine Ganxi goats (*n* = 9; 0 h control, 2 h/6 h transport groups) through an integrated experimental approach: TUNEL assays quantified apoptosis rates, immunohistochemistry mapped protein localization, Western blotting analyzed protein expression levels, and qPCR profiled gene expression of apoptotic regulators (Bax, Bcl-2) alongside autophagy-related markers (LC3B, p62, PINK1, Parkin).

**Results:**

Results indicated time-dependent cellular stress patterns, where the 2 h group displayed elevated apoptosis rates, while the 6 h group exhibited upregulated Parkin expression (*p* < 0.05) and altered regulation of apoptotic [Bcl-2-associated X-protein (Bax)/B-cell lymphoma-2 (Bcl-2)] and autophagy-related genes (Microtubule-associated protein 1 light chain 3B (LC3B), p62, PTEN-induced putative kinase 1 (PINK1)/Parkin). Protein localization analyses revealed compartment-specific responses, with Bcl-2/Bax primarily in bronchial epithelia and LC3B/PINK1/Parkin in alveolar cells, suggesting spatially distinct stress adaptation mechanisms. Observed molecular changes coincided with histological evidence of pulmonary alterations, implying a potential interplay between apoptosis and autophagy in transport-induced cellular stress. The identification of time-sensitive molecular shifts (e.g., transient apoptosis elevation at 2 h, and progressive Parkin activation at 6 h) could inform hypotheses for monitoring transport-associated physiological responses.

**Discussion:**

These findings highlight the need for further investigation into transport duration effects, with shorter intervals (e.g., ≤2 h) warranting evaluation for acute stress mitigation, and prolonged transport (e.g., >6 h) requiring characterization of cumulative autophagic impacts. The mechanistic insights can contribute to developing science-informed strategies for assessing transport stress, aligning animal welfare research with objectives to enhance sustainable livestock management practices.

## 1 Introduction

Transportation stress, driven by compounded oxidative, thermal, and physiological challenges, compromises animal welfare through multi-organ dysfunction and systemic physiological disturbances (1–3). Transport duration critically impacts animal welfare, as prolonged exposure disrupts homeostasis through thermoregulatory failure, antioxidant depletion, and immunosuppression, all of which are biomarkers of welfare compromise (4). Unmitigated stress not only elevates morbidity and mortality risks but can also exacerbate disease transmission susceptibility and meat quality deterioration (e.g., DFD/PSE defects), reflecting both ethical and economic implications for livestock systems (5–7). These welfare compromises extend beyond animal suffering, threatening public health security and sustainable food production, thereby underscoring the urgency for science-driven interventions to align transport practices with welfare-centric husbandry standards.

As a vital component of the respiratory system, the lungs primarily facilitate gas exchange while additionally contributing to drug metabolism and immune regulation (8, 9). Transportation stress induces pathological alterations in the caprine respiratory system, particularly affecting the structural integrity of pulmonary tissues, trachea, and bronchial structures (10). Extended transportation periods have been associated with the development of severe respiratory pathologies, including fibrinous pleuropneumonia and bovine respiratory disease complex (BRDC) (11–13). Emerging evidence suggest that bacterial endotoxins can induce pathological apoptosis in pulmonary epithelial cells through prolonged low-concentration exposure (14, 15). B-cell lymphoma-2 (BCL2)-associated X (Bax)-mediated apoptosis has been demonstrated to accelerate murine mortality and promote the pathogenesis of pulmonary disorders, including emphysema (16). Moderate apoptosis plays a dual physiological role, protecting against pulmonary infections and oncogenesis while preventing the accumulation of dysfunctional cells that may deplete metabolic resources and promote carcinogenesis or autoimmune disorders. This regulated cell death process facilitates recycling of cellular material and energy homeostasis (17–20). Apoptosis is primarily regulated through two distinct pathways: the receptor-mediated pathway and the mitochondrial pathway. The latter is controlled by the B-cell lymphoma-2 (Bcl-2) protein family, wherein Bcl-2 proteins inhibit apoptosis by antagonizing the pro-apoptotic function of Bax proteins (21–23). Cellular autophagy is a conserved catabolic process involving the sequestration of damaged organelles and long-lived proteins within autophagosomes for subsequent lysosomal degradation. Based on distinct cargo delivery mechanisms, autophagy is classified into three primary forms: macroautophagy, microautophagy, and chaperone-mediated autophagy (24, 25). Autophagy serves as a crucial cellular recycling mechanism, maintaining metabolic homeostasis and mitigating stress-induced damage. Notably, in mesenchymal progenitor cells, autophagy has a cytoprotective effect against bone marrow failure following severe intermittent stress (26–28). Recent genetic studies have established autophagy as a critical pathway in the pathogenesis of various human diseases, including neurodegenerative disorders, malignancies, inflammatory conditions, and autoimmune pathologies (29, 30). Excessive autophagy induces myocardial mitochondrial dysfunction and disrupts cellular homeostasis, contributing to both acute and chronic cardiac pathologies (31, 32). Core autophagy regulators, including Microtubule-associated protein 1 light chain 3B (LC3B), Sequestosome-1 (SQSTM1/p62), PTEN-induced putative kinase 1 (PINK1), and Parkin, mediate autophagic flux through interconnected signaling networks. Extensive research has revealed significant crosstalk between apoptotic and autophagic pathways, with their dynamic equilibrium maintaining metabolic homeostasis and modulating immune regulation (33–35). This study investigates the impact of transportation stress on apoptotic and autophagic pathways in caprine pulmonary cells by analyzing key regulatory proteins and genes, and provides mechanistic insights for developing preventive and therapeutic strategies against transportation-induced lung injury.

## 2 Materials and methods

### 2.1 Animals and experimental design

Nine healthy Ganxi male goats (*Capra hircus*) of similar body weight (13.89 ± 2.96 kg) and age (1 year) were selected for this study from Jiangxi Mulei Agriculture and Forestry Development Co., Ltd. (Ganxi Goat Breeding Farm). The selected goats were randomly divided into a control group, a 2 h transportation group, and a 6 h transportation group, with three goats in each group. The transportation groups were subjected to standard road conditions (4.2 × 2.2 × 1.8 m compartment, 35–45 km/h, and 28–32°C) with fasting protocols. The control group was not treated. After transportation, the animals were immediately euthanized using intravenous sodium pentobarbital (90 mg/kg), without rest periods. No transportation-related incidents occurred. Death was confirmed through cardiac auscultation. Euthanasia procedures were followed based on the Chinese Association for Laboratory Animal Sciences guidelines. All protocols were approved by Yichun University’s Animal Ethics Committee (License: JXSTUDKY2019009). Tissue samples were either fixed in 4% paraformaldehyde or snap-frozen in liquid nitrogen and stored at −80°C.

### 2.2 Preparation of paraffin sections

Paraffin-embedded lung tissue sections were prepared as detailed below. The samples were fixed in 4% paraformaldehyde and rinsed under 24 h running water. This was followed by dehydration using a graded ethanol series (70, 80, 90, 95, and 100%), clearing in xylene, and embedding in paraffin wax. Solidified blocks were sectioned at 5 μm thickness using a rotary microtome (RM2235, Leica Biosystems, Buffalo Grove, IL, United States). The sections were stored at 4°C for subsequent analysis.

### 2.3 TUNEL assay

Paraffin sections were routinely dewaxed, rehydrated, and immersed in distilled water and PBS (for 5 min each). TUNEL staining was performed using a TUNEL kit (MK1015, Wuhan Boster Biological Technology, Ltd., Wuhan, China). The sections were then incubated with Proteinase K (20 μg/mL in PBS, pH = 7.4) at 37°C for 20 min, followed by five PBS washes (5 min/wash). Test group samples were treated with 50 μL TUNEL reaction mixture, while negative controls were only labeled with 50 μL fluorescein-dUTP. The sections were incubated in a humidified dark chamber (37°C, 60 min) and washed three times in PBS (5 min/wash). Subsequently, 50 μL horseradish peroxidase (POD)-conjugated solution was applied under identical conditions (37°C, 30 min). After three PBS washes, color development was initiated with 50 μL DAB substrate (10 min, room temperature). TUNEL-positive apoptotic cells were identified by yellow/brownish-yellow nuclear staining with characteristic morphological changes (chromatin condensation, margination, fragmentation, or lysis), contrasting with blue-stained normal nuclei. Apoptosis rates were determined by counting positive cells in 10 randomly selected fields per section, viewed under 400 × magnification. The formula used for calculation is: (apoptosis-positive cells/total cells) × 100%.

### 2.4 Immunohistochemistry

**Figure 1:**
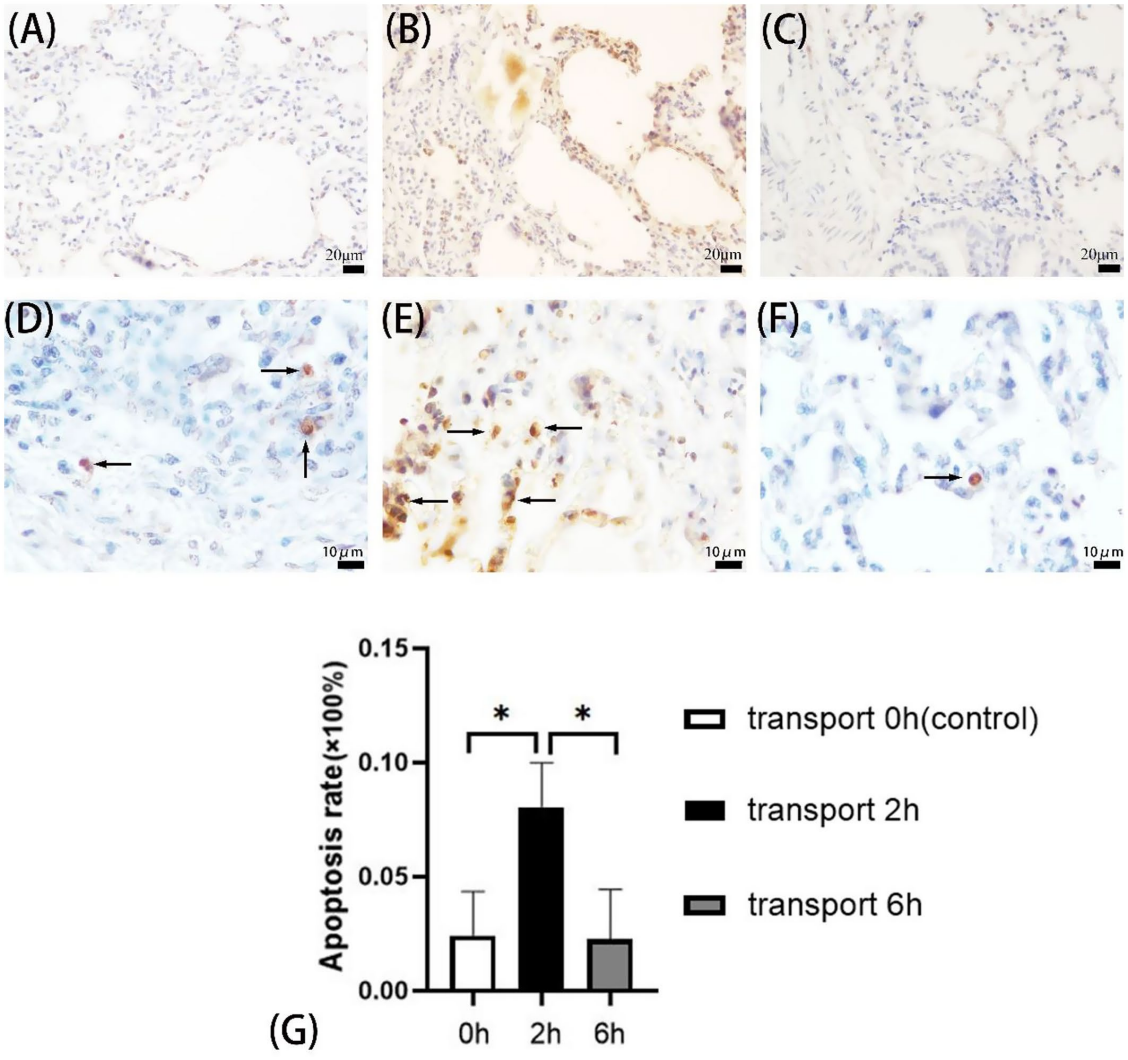
Apoptosis in goat lungs (TUNEL assay). **(A–C)** 400× microscope field of view, **(D–F)** 1,000× microscope field of view, **(A,D)** control group, **(B,E)** 2 h transport group, **(C,F)** 6 h transport group; the arrows show the TUNEL reaction positive cells, specifically manifested as yellow/brownish-yellow nuclear staining with characteristic morphological changes (chromatin condensation, margination, fragmentation, or lysis), contrasting with blue-stained normal nuclei. **(G)** Apoptosis rate in goat lung cells, the heights of the white, black, and gray columns in the bar chart correspond to the apoptosis rates of lung cells in goats in the control group, 2-h transportation group and 6-h transportation group, respectively. *Indicates significant difference (*P* < 0.05).

**Figure 2:**
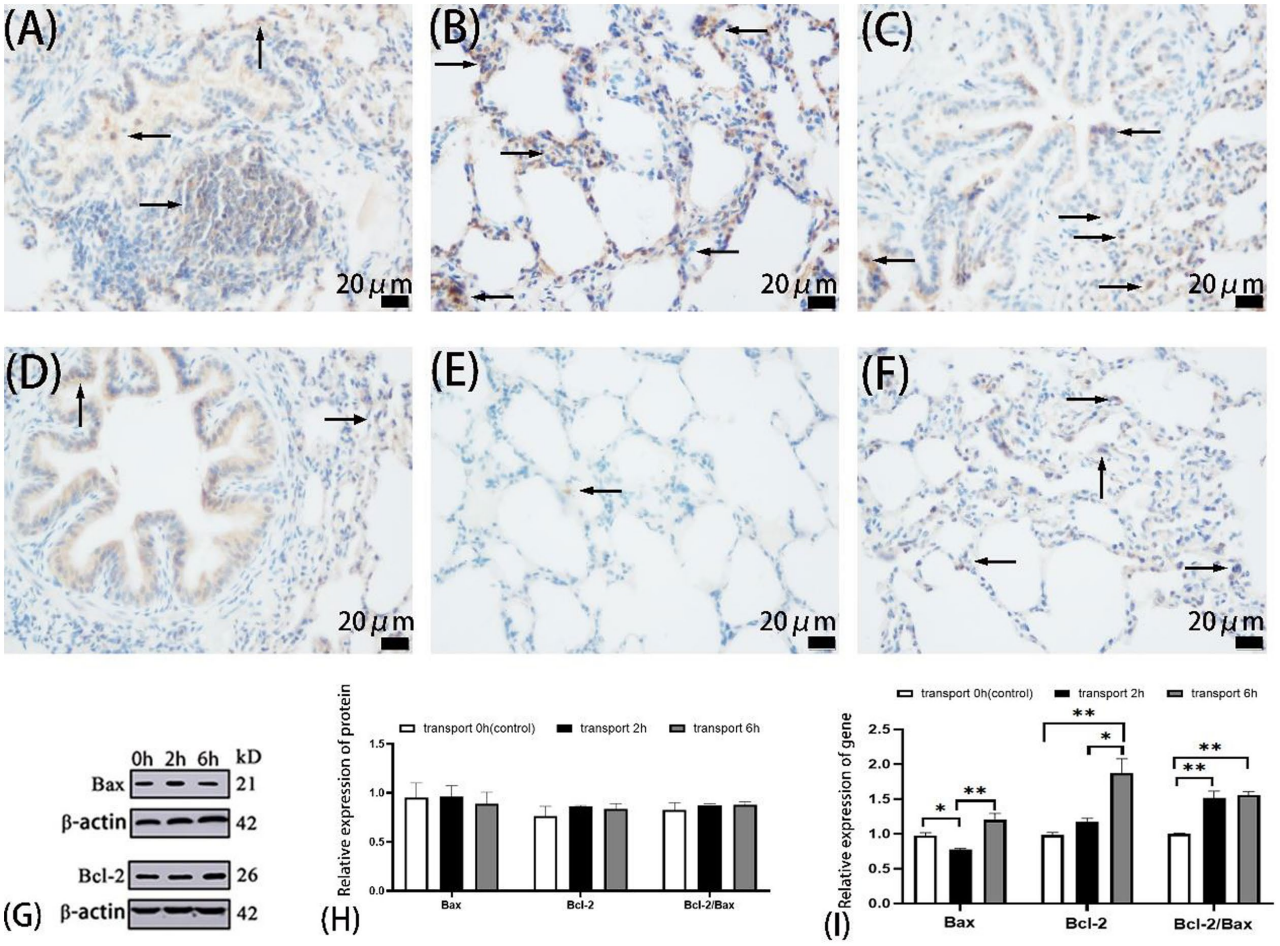
Apoptosis in goat lungs (immunohistochemistry, western blot and qRT-PCR). **(A–C)** The distribution of Bax protein. **(D–F)** The distribution of Bcl-2 protein. **(A,D)** Control groups, **(B,E)** 2 h transport groups, **(C,F)** 6 h transport groups; the positive expression regions are shown by arrows (immunohistochemistry 400×), immunoreactivity was graded as: strong (brown), moderate (yellow), weak (light yellow), or negative (no staining). **(G)** Protein blots of Bax and Bcl-2, the number at the right end of Protein Blots represents the molecular weight of the corresponding protein, while the upper 0 h, 2 h, and 6 h correspond to the Western blot results of the control group, 2-h transportation group and 6-h transportation group, respectively; **(H)** Expression of apoptosis-related proteins in goat lungs; **(I)** Expression of apoptosis-related genes in goat lung, the heights of the white, black, and gray bars in bars **(H,I)** correspond to the relative expression levels of proteins and genes in the lungs of goats in the control group, 2-h transportation group, and a 6-h transportation group, respectively. *Significant difference (*P* < 0.05), **highly significant difference (*P* < 0.01). Data are presented as the means ± SD and the internal reference gene is β-actin.

Paraffin sections were processed using a Rabbit Two-Step Assay Kit (PV-9001, Beijing Zhongshan Jinqiao Co., Ltd., Beijing, China) following the manufacturer’s instructions. The sections were treated thrice with xylene (for 10 min each), dehydrated with a gradient of alcohol, rinsed for 1 min with distilled water, and then immersed in PBS buffer. Antigen retrieval was performed using 10 mM sodium citrate buffer (pH 6.0) with microwave heating, followed by washing with PBS three times (3 min/wash). Endogenous peroxidase activity was blocked with 3% H_2_O_2_ (37°C, 10 min). Primary antibodies were applied overnight at 4°C (negative controls received PBS only). Following overnight incubation, the sections were treated with enhancement solution (37°C, 20 min), washed with PBS, and incubated with enzyme-conjugated anti-rabbit IgG polymer (37°C, 20 min). After additional PBS washes, color development was performed using the DAB kit (ZLI-9018, Beijing Zhongshan Jinqiao Co., Ltd., Beijing, China) for 7 min at room temperature under light-protected conditions. The sections were sequentially rinsed with tap and distilled water, counterstained with hematoxylin (8 s), differentiated in acid alcohol (1 s × 3), dehydrated, cleared, and mounted. Immunoreactivity was graded as: strong (brown), moderate (yellow), weak (light yellow), or negative (no staining). Scale bars (20 μm) were uniformly positioned in the lower-right corner of all histological images (Figures 1A–F, 2A–L) without obstructing positively stained regions. The primary and secondary antibodies used in this section are shown in Tables 1, 2, respectively.

**Table 1:** Information on the primary antibodies used in this study.

Name	Host/subtype	Dilution ratio of immunohistochemistry	Dilution ratio of Western blot	Origin	Product number
BAX Polyclonal antibody	Rabbit/IgG	1:500	1:1000	Proteintech Group, Inc. (Wuhan, China)	50599-2-Ig
Bcl2 Polyclonal antibody	Rabbit/IgG	1:200	1:500	Proteintech Group, Inc. (Wuhan, China)	26593-1-AP
Anti-LC3B	Rabbit/IgG	1:400	1:11000	Wuhan Boster Biological Technology, Ltd. (Wuhan, China)	PA01524
P62 Rabbit pAb	Rabbit/IgG	1:100	1:1500	Bioss (Beijing, China)	bs-2951R
PINK1 Rabbit pAb	Rabbit/IgG	1:100	1:1500	Bioss (Beijing, China)	bs-22173
Parkin Rabbit pAb	Rabbit/IgG	1:300	1:1000	Bioss (Beijing, China)	bs-23687R

**Table 2:** Information on the secondary antibodies used in this study.

Name	Host/isotype	Dilution ratio	Origin	Product number
Goat Anti-Mouse IgG H&L (HRP)	Goat/IgG1	1:5000	Abcam (Shanghai, China)	ab6789
Anti-Beta Actin	Mouse/IgG1	1:10000	Wuhan Boster Biological Technology, Ltd. (Wuhan, China)	BM0627

### 2.5 Western blot analysis

**Figure 3:**
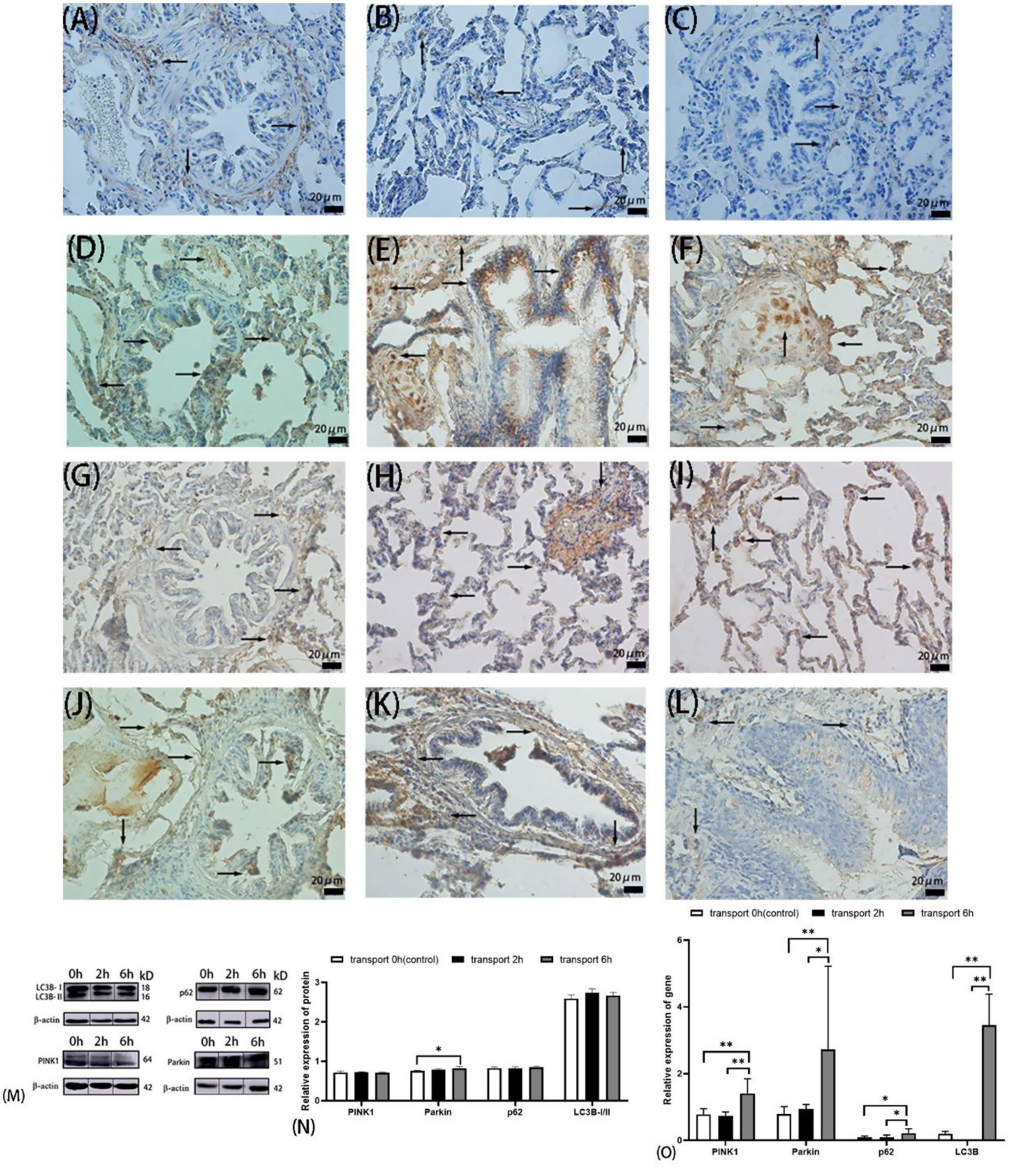
Autophagy in goat lungs (immunohistochemistry, western blot, and qRT PCR). **(A–C)** The distribution of LC3B protein. **(D–F)** The distribution of p62 protein. **(G–I)** The distribution of PINK1 protein. **(J–L)** The distribution of Parkin protein; **(A,D,G,J)** control groups; **(B,E,H,K)** 2 h transport groups; **(C,F,I,L)** 6 h transport groups; positive expression is shown by the arrows in the figure, immunoreactivity was graded as: strong (brown), moderate (yellow), weak (light yellow), or negative (no staining). **(M)** Protein bands of PINK1, Parkin, p62, LC3B-I, and LC3B-II, the number at the right end of Protein Blots represents the molecular weight of the corresponding protein, while the upper 0 h, 2 h, and 6 h correspond to the Western blot results of the control group, 2-h transportation group, and a 6-h transportation group, respectively; **(N)** Expression of PINK1, Parkin, p62, and LC3B-I/II protein; **(O)** Expression of PINK1, Parkin, p62, and LC3B gene, the heights of the white, black, and gray bars in bars **(N,O)** correspond to the relative expression levels of proteins and genes in the lungs of goats in the control group, 2-h transportation group and 6-h transportation group, respectively. *Significant difference (*P* < 0.05), **highly significant difference (*P* < 0.01). Data are presented as the means ± SD and the internal reference gene is β-actin.

Frozen lung tissue samples (50 mg) were homogenized in 1 mL lysis buffer on ice using a tissue homogenizer. Then, 500 μL of the homogenate was transferred to a 1.5 mL microcentrifuge tube, mixed with 1 mL extraction reagent, and incubated at 4°C for 10 min. After centrifugation at 10,000 × g (4°C, 10 min), the supernatant and lower phase were discarded to retain the intermediate protein membrane. The pellet was air-dried at room temperature with the tube caps open. The dried precipitate was resuspended in an appropriate volume of 2% SDS solution, boiled at 95°C for 10 min, and equilibrated at room temperature for 30 min. The protein supernatant was collected by centrifugation at 12,000 × g for 5 min. Protein concentrations were determined using a BCA assay kit (P1250, Applygen Technologies Inc., Beijing, China), and equal amounts were added to protein loading buffer and were determined using a BCA assay kit (AR0146, Boster Biological Technology, Wuhan, China) following the manufacturer’s instructions, then mixed with protein loading buffer for later use. Samples were mixed with 4 × buffer, denatured (95°C, 5 min), and separated by SDS-PAGE (10% separating gel, 5% stacking gel) for 2 h. Proteins were transferred to 0.45 μm PVDF membranes (IPVH00010, Beijing Qiangxin Biorepublic Co., Ltd., Beijing, China) for 75 min. The membranes were blocked with 5% skim milk for 2 h, and washed with TBST (5 min × 3). Subsequently, the protein membrane was incubated overnight with the primary antibody, with gentle, continuous flipping during the process. After three TBST washes (for 10 min each), the membranes were incubated with secondary antibodies (diluted in 2% skim milk; see Table 2) for 2 h at room temperature and then washed thrice with TBST. Protein bands were detected using an ECL kit (P10100, Suzhou Xinsaimei Biotechnology Co., Ltd., Suzhou, China) using a 1:1 mixed A/B solution and imaged with a chemiluminescence system (Amersham Imager 600, GE Healthcare, United States). Quantitative analysis was performed using Image Pro Plus 6.0 (Media Cybernetics, United States). Relative expression levels were normalized to β-actin. All the experiments were independently repeated three times. Protein markers, molecular weights, and experimental conditions were annotated for clarity. As seen in Figure 3M, separator lines were inserted between samples to distinguish different experimental conditions and improve visual presentation, without obscuring protein expression patterns.

### 2.6 Real-time fluorescence quantitative PCR

Total RNA was extracted using an HP Total RNA Kit (R6812, United States Omega Bio-Tek (Guangzhou), Guangzhou, China) and quantified spectrophotometrically. Reverse transcription was performed using HiScript II QRT SuperMix (10 μL reaction volume), with cDNA diluted in 20 μL ddH2O for subsequent qPCR analysis. Gene-specific primers were designed using Premier 6.0 software based on caprine reference sequences: Bcl-2 (NM001314213.1), Bax (XM018062750.1), and β-actin (NM001314342.1). Primers were designed using Primer Premier 6.0 (Premier Biosoft, Canada) software and sent to Guangzhou Huada Gene Medical Laboratory Co., Ltd. for synthesis. The primer sequences used in the experiment are shown in Tables 3, 4. qPCR was performed using ChamQ SYBR qPCR Master Mix (Q311-02, Vazyme Biotech Co., Ltd., Nanjing, China) in 10 μL reactions, containing 5 μL 2 × SYBR Primix Ex Taq II, 1 μL each of 4 μmol/L primers, 2.8 μL cDNA, and 0.2 μL 50 × ROX Reference Dye. Reactions were conducted on an ABI 7500 system with the following protocol: 95°C for 3 min; 40 cycles at 95°C for 10 s and at 68°C for 30 s. Relative expression was calculated using the 2-ΔΔCt method.

**Table 3:** Sequences of qPCR primers for autophagy-related proteins.

Gene	Primer sequence (5′-3′)
LC3B	F: AGAAGGCGCTTACAGCTCAATGC
	R: ACTTCACAAATCGGGAGTGGGACAC
P62	F: AGGTGCCCCGAAATATGGTG
	R: GCGGAGCACAGGTCATAGTC
PINK1	F: TCATCCAGCGAAGCCATCTTTAGC
	R: TCCCTTGGGTCTTCCGTGAGTG
Parkin	F: GCATAACGTGTACGGACATCAGGAG
	R: CAGGTGGAAGCAGTCTAAGCAGATC
β-actin	F: CTCTTCCAGCCTTCCTTCCT
	R: GGGCAGTGATCTCTTTCTGC

**Table 4:** Sequences of qPCR primers for apoptosis-related proteins.

Gene	Primer sequence (5′-3′)
Bax	F: GGCCCTTTTGCTTCAGGGTT
	R: CAGACACTCGCTCAGCTTCT
Bcl-2	F: GAGTTCGGAGGGGGTCATGTG
	R: TACAGCTCCACAAAGGCGTC
β-actin	F: CTCTTCCAGCCTTCCTTCCT
	R: GGGCAGTGATCTCTTTCTGC

### 2.7 Statistical analysis

Statistical analysis was performed using SPSS 26.0 to compare protein and gene expression across transport durations. Parametric data from different groups were compared using one-way ANOVA, followed by the least significant difference (LSD) test. Significance levels were defined as: *p < 0.05, **p < 0.01. Data are presented as mean ± standard deviation (SD). Graphical representations were generated using GraphPad Prism 8.0.

## 3 Results

### 3.1 Effects of transportation stress on apoptosis and expression of Bax and Bcl-2 in goat lung cells

#### 3.1.1 Apoptosis in goat lung cells

TUNEL assays revealed pulmonary apoptosis in both control and transported goats (Figures 1A–F). The 2 h transport group exhibited significantly higher apoptosis rates compared to both 6 h transport and control groups (*p* < 0.05), while no significant difference was observed between the control and 6 h groups (*p* > 0.05) (Figure 1G).

#### 3.1.2 Localization of apoptosis-related proteins Bax and Bcl-2 in the lungs

Immunohistochemical analysis revealed similar Bax localization patterns across groups, predominantly in alveolar epithelial cytoplasm, bronchiolar epithelium, and select alveolar septal cells (Figures 2A–F). Bax expression was predominantly weak-to-moderate in the control and 6 h groups, while strong-to-moderate in the 2 h group. Bcl-2 exhibited similar cellular distribution but differential expression intensity: weak-to-moderate in the control and 6 h groups, contrasting with minimal expression in the 2 h group.

#### 3.1.3 Expression of Bax and Bcl-2 proteins in goat lungs

Western blot analysis revealed no significant differences (*p* > 0.05) in Bcl-2, Bax expression, or Bcl-2/Bax ratios among the control, 2 h, and 6 h transport groups (Figure 2H).

#### 3.1.4 Bax and Bcl-2 gene expression in goat lungs

qPCR analysis (Figure 2I) demonstrated significantly reduced Bax expression in the 2 h group compared to the control (*p* < 0.05) and 6 h groups (*p* < 0.01). Bcl-2 expression was significantly elevated in the 6 h group in comparison to the control and 2 h groups (*p* < 0.01), with no difference between the 2 h and control groups (*p* > 0.05). The Bcl-2/Bax ratio was significantly increased in both the transport groups compared to the controls (*p* < 0.01).

### 3.2 Effects of transportation stress on autophagy and expression of LC3B, p62, PINK1, and Parkin in goat lung cells

#### 3.2.1 Expression and localization of autophagy-related proteins LC3B, p62, PINK1, and Parkin in goat lungs

Immunohistochemical analysis revealed moderate to strong cytoplasmic expression of all four autophagy-related proteins in alveolar epithelial cells (Figures 3A–L). LC3B, p62, and PINK1 exhibited similar localization patterns across the control and transported groups. LC3B was predominantly expressed in the cytoplasm of alveolar, bronchiolar, and arteriolar epithelial cells, with notably stronger brownish staining in the 2 h transport group compared to the control and 6 h transport groups. p62 showed no positive expression in the alveolar septa or terminal bronchiolar epithelial cells but displayed extensive, strong brown staining in the hyaline cartilage of small bronchiolar outer membranes (Figures 3E,F). The autophagy-related proteins PINK1 and Parkin were localized in both the nucleus and cytoplasm. Parkin expression in the 2 h and 6 h transport groups was widely distributed in the lungs, exhibiting light yellow or yellow staining, except in the alveolar septa. In contrast, Parkin expression in the control group and PINK1 expression across all groups were nearly absent in the bronchial regions, also referred to as the conductive portion of the lungs (Figures 3G–L).

#### 3.2.2 Expression of LC3B, p62, PINK1, and Parkin proteins in goat lungs

Western blot analysis of autophagy-related proteins (Figure 3N) revealed no significant differences in the relative expression of p62, LC3B, or the LC3B-I/LC3B-II ratio among the control, 2 h transport, and 6 h transport groups (*p* > 0.05). However, Parkin expression was significantly upregulated in the 6 h transport group compared to the control (*p* < 0.05).

#### 3.2.3 Expression of LC3B, p62, PINK1, and Parkin genes in goat lungs

qPCR analysis (Figure 3O) revealed that LC3B gene expression was significantly higher in the control group compared to the 2 h and 6 h transport groups (*p* < 0.01). In contrast, p62, PINK1, and Parkin gene expressions were significantly upregulated in the 6 h transport group (*p* < 0.05), with PINK1 and Parkin showing highly significant increases compared to the control group (*p* < 0.01). No significant differences were observed in p62, PINK1, or Parkin gene expressions between the control and 2 h transport groups (*p* > 0.05).

## 4 Discussion

Transportation exposes animals to compounded environmental stressors, including thermal discomfort, mechanical vibrations, and toxin exposure, which compromise their welfare by driving oxidative and thermal stress responses, as evidenced by elevated markers (Malondialdehyde, Reactive oxygen species, and DNA/RNA oxidative stress damage products) and upregulated heat shock proteins (HSP27, 70, 90) in goats (36–39). These disruptions exhibit pulmonary tropism, with respiratory dysfunction directly compromising welfare through impaired gas exchange and barrier function. Crucially, transport-induced immunosuppression and pulmonary damage facilitate pathogenic invasion, increasing susceptibility to severe respiratory pathologies, such as Bartonella pneumonia and acute respiratory distress syndrome (ARDS)—conditions directly linked to animal suffering and productivity losses (13, 40–42). Structural alterations in bronchial tissues and oxidative modulation of apoptotic (Bcl-2/Bax) and autophagic mediators (LC3B, PINK1, and Parkin) further underscore the molecular basis of transport-related welfare compromises (10, 43). The interplay of physical injury, inflammation, and stress-mediated cell death pathways highlights systemic welfare threats, with preclinical evidence suggesting apoptosis attenuation via Bcl-2/Bax modulation as a potential welfare-protective strategy (44, 45). By mapping these molecular cascades to organ-level dysfunction, this study identifies biomarkers (e.g., Heat shock proteins and oxidative markers) that could inform welfare-centric transport protocols, balancing ethical husbandry with sustainable livestock management.

Our findings demonstrate that transportation stress induces pulmonary apoptosis in goats, with TUNEL assays revealing significantly elevated apoptosis rates following 2 h of transport. Interestingly, the 6 h transport group exhibited apoptosis rates comparable to the controls, indicating a net reduction in apoptotic cells between 2 h and 6 h of transport. The observed apoptotic cell reduction suggests an intrinsic anti-stress mechanism that simultaneously inhibits apoptosis and facilitates apoptotic cell clearance in caprine pulmonary tissues during prolonged transport. Existing evidence demonstrates extensive crosstalk between stress-induced apoptotic and autophagic pathways. Autophagy and apoptosis share common inducers and exhibit bidirectional regulation, where autophagy prevents apoptosis by eliminating damaged mitochondria and associated death signals, while mitochondrial release of cytochrome c during autophagy can activate caspase-mediated apoptosis. Furthermore, PINK1 and Parkin modulate apoptotic signaling through Bax and p53 regulation. Apoptotic regulators, including p53 and Bcl-2 family proteins, modulate mitochondrial membrane stability, thereby influencing mitophagy. Additional crosstalk mediators encompass ERLIN and Beclin1, among others (33, 46, 47). The apoptotic–autophagic crosstalk is critically regulated by calcium-mediated signaling pathways (35, 48). qPCR analysis revealed significant upregulation of the Bcl-2/Bax ratio following 2 h transport, while LC3B, p62, PINK1, and Parkin expression increased after 6 h transport. These findings indicate time-dependent autophagosome formation and ubiquitin-mediated mitophagy activation in response to transportation stress. The concurrent upregulation of anti-apoptotic Bcl-2 and autophagy-related genes with prolonged transport duration demonstrates functional crosstalk between apoptotic and autophagic pathways in the studied cellular system. This regulatory mechanism likely represents stress-induced apoptosis triggering mitophagy, thereby establishing a cytoprotective feedback loop in caprine pulmonary cells. Apoptotic cell clearance involves a coordinated mechanism combining classical phagocytosis, autophagic processes, and alveolar macrophage-mediated efferocytosis within the pulmonary mucosal and interstitial compartments. Crucially, autophagy maintains cellular ATP homeostasis, thereby facilitating efficient apoptotic cell clearance through these coordinated mechanisms (33, 49, 50). TUNEL assays indicated rapid apoptosis induction within 0–2 h of transport, coinciding with stable autophagy-related gene expression. This temporal pattern suggests that transport-induced DNA damage or death receptor activation triggers immediate apoptosis, potentially suppressing concurrent autophagic responses during this amplification phase. However, the temporal relationship between stress-induced apoptosis and autophagy requires further investigation (35). Notably, baseline apoptosis was observed in non-transported controls, indicating constitutive expression of apoptotic regulators Bax and Bcl-2 in caprine pulmonary epithelium. Their balanced interaction maintains epithelial cell population homeostasis.

Immunohistochemical analysis revealed comparable Bax and Bcl-2 expression patterns across control and transport groups, with predominant localization in alveolar epithelium, bronchiolar epithelium, and select alveolar septa. Most cells in the controls and 6 h transport groups exhibited weak-to-moderate Bax/Bcl-2 expression. In contrast, the 2 h group showed strong-to-moderate Bax expression with minimal Bcl-2 detection, correlating with elevated apoptosis rates through altered Bcl-2/Bax expression dynamics. All four autophagy markers demonstrated positive alveolar epithelial expression, with LC3B, p62, and PINK1 exhibiting consistent cellular localization patterns across control and transport groups. LC3B exhibited predominant cytoplasmic localization in alveolar, bronchiolar, and arteriolar epithelial cells, while p62 demonstrated intense immunoreactivity in bronchiolar hyaline cartilage. PINK1 and Parkin demonstrated nuclear and cytoplasmic localization. Parkin exhibited moderate pulmonary expression (excluding alveolar septa) in transport groups, while both Parkin in controls and PINK1 showed minimal bronchiolar expression across all airway levels (Figures 2G,J). Immunohistochemical analysis revealed transport stress-induced apoptosis and autophagy in specific pulmonary regions. Previous studies have demonstrated similar stress-mediated apoptosis in caprine immune organs (intestinal epithelium, spleen, and lymph nodes), resulting in immunosuppression and subsequent pulmonary macrophage depletion (16, 49, 50). Transport stress induces cardiac enzyme accumulation and mitochondrial dysfunction, promoting excessive mitophagy. Notably, mitochondrial complex I in pulmonary epithelium is crucial for lung development, indicating that integrated mitochondrial stress responses determine epithelial cell fate (51, 52). In summary, transport-induced apoptosis compromises pulmonary integrity through dual mechanisms: direct pathogen-mediated alveolar-capillary barrier disruption and indirect structural–functional impairment via immunosuppression, collectively impairing respiratory and immune functions.

Western blot analysis revealed comparable expression levels of Bcl-2, Bax, LC3B, PINK1, and p62 across all experimental groups. Parkin expression was significantly elevated in the 6 h transport group compared to the controls. The discordance between gene and protein expression patterns of Bax and Bcl-2 in transport groups suggests a temporal lag, where rapid transcriptional activation precedes protein translation completion. Similarly, the observed gene-protein expression discrepancy in autophagy markers (LC3B, p62, PINK1, and Parkin) likely reflects differential kinetics between transcriptional activation and protein synthesis. Notably, similar gene–protein expression discrepancies have been observed in caprine spleen, lymph nodes, and intestinal tissues following transport stress, suggesting a systemic temporal dissociation between transcriptional and translational regulation of apoptotic markers. This phenomenon reflects delayed protein synthesis kinetics relative to transcriptional activation (7). However, tissue-specific variations in gene-protein expression correlations likely reflect molecular and cellular context-dependent regulatory mechanisms.

Predominant Bax expression during initial transport phases was revealed by qPCR analysis, indicating early apoptotic activation. Following 2 h and 6 h transport, pulmonary Bcl-2/Bax ratios significantly increased compared to the controls, demonstrating time-dependent predominance of anti-apoptotic signaling. Notably, autophagy-related genes (LC3B, p62, PINK1, and Parkin) exhibited significant upregulation following 6 h transport, coinciding with reduced apoptotic activity. These observations indicate a potential mechanism whereby stress-induced apoptosis can initiate ubiquitin-dependent mitophagy, which can subsequently reduce apoptotic risk through clearance of mitochondria containing death signals, potentially offering cytoprotection in caprine pulmonary cells. Previous studies have demonstrated context-dependent protective roles of autophagy against cell death. For instance, reticulum-mediated autophagy mitigates PM2.5-induced endothelial apoptosis, while cadmium-exposed trophoblasts exhibit p62-dependent caspase-9 degradation through autophagy activation. Notably, cadmium-induced autophagy appears apoptosis-dependent, with autophagy-related gene 4B (Atg4B)-Bcl-2 interactions potentially modulating apoptotic-autophagic crosstalk via Bcl-2-Beclin1 dissociation (46, 53, 54). Additional stress-responsive mechanisms may contribute to cellular protection, including the nuclear factor-erythroid 2-related factor 2 pathway-mediated regulation of oxidative stress markers (Reactive oxygen species, Heat shock proteins, Nitric oxide), which collectively exert antioxidant and anti-apoptotic effects (29, 55). The elevated apoptosis rate in the 2 h transport group, contrasting with gene expression patterns, may reflect delayed clearance of stress-induced apoptotic cells by pulmonary scavenging mechanisms. This temporal pattern, coupled with early Bax downregulation in the 2 h group, suggests rapid and intense apoptotic activation during initial transport phases in caprine pulmonary cells.

The interplay between autophagy and apoptosis critically influences pulmonary health, with direct implications for animal welfare under transport stress. In fibrotic lung diseases (idiopathic pulmonary fibrosis, cystic fibrosis, and silicosis), autophagy modulates disease progression through fibroblast apoptosis regulation and epithelial cell senescence, while dysregulated macrophage autophagy exacerbates fibrosis mechanisms, underscoring the delicate balance required to maintain respiratory welfare (44, 56, 57). Similarly, preterm neonates with bronchopulmonary dysplasia exhibit oxidative stress-driven programmed cell death pathways (Notch4/SIRT1/P53/Bax, RIPK3/NF-κB) that impair alveolar development, paralleling transport stress scenarios where pulmonary structural integrity is compromised (58, 59). The mitochondrial PINK1/Parkin pathway’s role in attenuating inflammation and apoptosis further highlights potential therapeutic targets to mitigate welfare risks (46, 60). Critically, prolonged transport stress disrupts the apoptosis–autophagy equilibrium, with excessive apoptosis threatening pulmonary homeostasis and respiratory function, which is a welfare concern linked to impaired physiological resilience. Conversely, stress-induced autophagy may serve as an adaptive mechanism to counterbalance apoptotic cell death (61–63). This study demonstrates that transport stress induces apoptosis and autophagy in lung cells. Significant alterations in the expression of apoptosis-related genes (Bax and Bcl-2) and autophagy-related genes (LC3B, p62, PINK1, and Parkin) were observed in goat lungs following varying transport durations, highlighting their roles in transport stress-mediated apoptosis and autophagy. These findings emphasize the need to optimize transport durations and conditions to preserve pulmonary autophagy–apoptosis crosstalk, thereby safeguarding respiratory welfare in livestock systems.

## Data Availability

The original contributions presented in the study are included in the article/supplementary material, further inquiries can be directed to the corresponding authors.
